# Improving Aboriginal maternal and infant health services in the ‘Top End’ of Australia; synthesis of the findings of a health services research program aimed at engaging stakeholders, developing research capacity and embedding change

**DOI:** 10.1186/1472-6963-14-241

**Published:** 2014-06-02

**Authors:** Lesley Barclay, Sue Kruske, Sarah Bar-Zeev, Malinda Steenkamp, Cathryn Josif, Concepta Wulili Narjic, Molly Wardaguga, Suzanne Belton, Yu Gao, Terry Dunbar, Sue Kildea

**Affiliations:** 1 University Centre for Rural Health, North Coast, Sydney Medical School, University of Sydney, Sydney, NSW, Australia; 2 Queensland Centre for Mothers and Babies, University of Queensland, Brisbane, Queensland, Australia; 3 School of Nursing and Midwifery, Flinders University, Adelaide, South Australia, Australia; 4 Wadaye Post Office, via, Winellie, Northern Territory, Australia; 5 Midwifery Research Unit, Australian Catholic University and Mater Research Women’s Health and Newborn Services, Brisbane, Queensland, Australia; 6 Menzies School of Health Research, Darwin, Northern Territory, Australia; 7 School of Indigenous Knowledges and Public Policy, Charles Darwin University, Darwin, Northern Territory, Australia

**Keywords:** Aboriginal, Australian, Indigenous maternity care, Indigenous infant care, Cultural safety, Indigenous health care workforce, Remote health services

## Abstract

**Background:**

Health services research is a well-articulated research methodology and can be a powerful vehicle to implement sustainable health service reform. This paper presents a summary of a five-year collaborative program between stakeholders and researchers that led to sustainable improvements in the *maternity services for remote-dwelling Aboriginal women and their infants in the Top End (TE) of Australia*.

**Methods:**

A mixed-methods health services research program of work was designed, using a participatory approach. The study area consisted of two large remote Aboriginal communities in the Top End of Australia and the hospital in the regional centre (RC) that provided birth and tertiary care for these communities. The stakeholders included consumers, midwives, doctors, nurses, Aboriginal Health Workers (AHW), managers, policy makers and support staff. Data were sourced from: hospital and health centre records; perinatal data sets and costing data sets; observations of maternal and infant health service delivery and parenting styles; formal and informal interviews with providers and women and focus groups. Studies examined: indicator sets that identify best care, the impact of quality of care and remoteness on health outcomes, discrepancies in the birth counts in a range of different data sets and ethnographic studies of ‘out of hospital’ or health centre birth and parenting. A new model of maternity care was introduced by the health service aiming to improve care following the findings of our research. Some of these improvements introduced during the five-year research program of research were evaluated.

**Results:**

Cost effective improvements were made to the acceptability, quality and outcomes of maternity care. However, our synthesis identified system-wide problems that still account for poor quality of infant services, specifically, unacceptable standards of infant care and parent support, no apparent relationship between volume and acuity of presentations and staff numbers with the required skills for providing care for infants, and an ‘outpatient’ model of care. Services were also characterised by absent Aboriginal leadership and inadequate coordination between remote and tertiary services that is essential to improve quality of care and reduce ‘system-introduced’ risk.

**Conclusion:**

Evidence-informed redesign of maternity services and delivery of care has improved clinical effectiveness and quality for women. However, more work is needed to address substandard care provided for infants and their parents.

## Background

Just over 500,000 Aboriginal and Torres Strait Islander people (hereafter referred to as Aboriginal) live in Australia today comprising fewer than three per cent of the Australian population [1]. Although many Aboriginal Australians have a good standard of living, too many remote living people experience unacceptable levels of disadvantage in living standards, life expectancy, education, health and employment [1]. Current data shows a wide ‘gap’ between the Australian Aboriginal and non- Aboriginal populations. Australia has committed to ‘Closing the Gap’ between Aboriginal and other Australians [2]. Maternal and infant health (MIH) is a particular area of concern with the perinatal mortality rate of Aboriginal infants 50% higher than for non-Aboriginal infants [3]. Similarly, rates of preterm birth and low birth weight (LBW) Aboriginal babies are nearly double those of non- Aboriginal Australians, signifying the need for serious and costly health care in the short-term, and foreshadowing long-term health problems, particularly in relation to chronic diseases [4]. Early antenatal care (ANC) and delivery of smoking cessation programs are linked to improved outcomes and are both significantly lower for Aboriginal women [2]. For all Australians, MIH outcomes worsen with increasing remoteness [5,6] where providing services is more challenging [2]. A greater proportion of Aboriginal births occur to women living in areas considered remote or very remote – 26% compared to two per cent of non- Aboriginal births [7]. Also, in the context of overall perinatal disadvantage for Aboriginal mothers, remoteness is associated with poorer infant outcomes [8].

This paper synthesises the findings of a program of linked concurrent research projects funded by the Australian National Health and Medical Research Council (NHRMC) and the Australian Research Council (ARC). These projects all shared a common goal which was to: 1) provide evidence to influence policy and service design; 2) contribute to sustained improvement though translation of the research and, by so doing; 3) improve the quality of health care for this population group [9]. A subsidiary goal (4) was to improve capacity through research training in a region where this had been limited.

Both funded studies were conducted in the same two remote communities of the Northern Territory (NT). Eighty percent of the NT Aboriginal population lives remotely [10] and two-thirds of Aboriginal births originate from these remote settings [11].

The synthesis of the two NHMRC and ARC projects as one program of work answers the overarching question: ‘How can we improve MIH services for remote-dwelling Aboriginal women and their infants?’

In all of our studies, we prioritised Aboriginal ethics [12] and principles [13] within the research. Where relevant, community leadership was provided by Aboriginal co-researchers. Ethical approval was obtained from the Human Research Ethics Committee of the Northern Territory Department of Health and the Menzies School of Health Research.

## Methods

### Setting and study participants

The Australian Research Council (ARC) funded research used social sciences techniques with small groups of women in both communities with an aim to investigate the experience of women and families with the health system and the process of their parenting.

The National Health and Medical Research Council (NHMRC) funded research used mixed-methods sub-studies and a participatory approach into health service design and delivery of MIH care for remote living Aboriginal women. This study, called ‘*1 + 1 = A healthy start to life’*, focused on health services in the year before (during pregnancy) and year after birth (1 + 1) to promote ‘a healthy start to life’. The participatory approach ensured engagement of the Aboriginal communities and industry stakeholders. Aboriginal women, policy makers, managers and clinicians helped guide the research. The NHMRC research comprised several quantitative and qualitative sub-studies investigating aspects of MIH including: health service organisation and utilisation; health outcomes for mothers and their infants; the quality and usefulness of routinely collected perinatal data; and the staff’s experience working in the system and their adherence to evidence based guidelines.

The majority of the non-epidemiological sub-studies were located in two purposively selected remote Aboriginal communities (Community ‘A’ and Community ‘B’) both of approximately 2,000-3,000 people in the Top End. Each is located approximately 500 km from the regional centre (RC). The RC has a hospital that provides tertiary, maternity, newborn and paediatric care, where some observational work and interviews were also conducted. Government health centre staff provided the antenatal care (ANC) in the remote communities with additional clinical support from outreach services. Women were routinely transferred to the RC at 38 weeks gestation to await birth while they lived in a hostel and received hospital based ANC. During this time multiple care providers, typically unknown to the women, attended them. After discharge, women and their infants were transferred back to their community.

For all studies, we built in methodological flexibility. This is consistent with a mixed-methods approach and allowed us to add new research questions based on findings early in the research [14]. Each sub-study addressed specific questions detailed in each successful research application to ARC or NHMRC and has been published, or is currently in the process of being published.

### Design and data collection

#### ***The sub-studies***

Each sub-study within the program of work funded by both ARC and NHMRC was conceptualized, designed, undertaken and reported within the overriding goal of informing improvements of services [15]. Table 1 outlines the detail from the sub-studies and describes the translation impact of the work and how ‘training’ opportunities were built in.

**Table 1:** Translation; dissemination and training associated with sub-studies conducted

**Sample, data used**	**Translation**	**Training/capacity development**
**Baseline data study:** Hospital and health centre data of 412 mothers and their 413 infants (2004–6) were audited from the two remote study sites and RC hospital; 120 hours of observation of maternal and infant health services in 3 settings and 60 semi-structured interviews with key stakeholders;	This work was instrumental in: developing the MGP and improvements to infant health care; informing the choice of indicators; well disseminated through publications.	PhD student A; thesis accepted December 2013; 5 publications.
**Epidemiological studies:** 7,560 mothers with singleton pregnancies from NT perinatal data using uni- and multivariate analyses for groupings by Aboriginal status; region (Top End/Central Australia); Remote/Urban residence; and the two field sites; 2,421 Aboriginal mothers aged 13–34 years and 254 non-Aboriginal teenagers with singleton pregnancies; Comparison of community records, birth registrations and perinatal counts in standardised data sets and;42 indicators sets with more than 1,000 individual indicators reviewed to develop core indicators for remote. Iterative Input from 30 experts as the list was developed.	*Confirmed that quality of care and remoteness, rather than Aboriginality,* influence the worse outcomes of remote living Aboriginal women and infants. We also demonstrated that *Aboriginality, rather than age, is the important variable* related to outcomes for young women. *The comparison* work showed errors in birth counts. that have significant influence on managers who should try to staff by volume and acuity of work but do not. The final study has potential to provide *targeted indicators* for remote living Aboriginal women and infants in the TE.	PhD student B graduated with 4 + 1 under review publications.
	We were able to differentiate subgroups of Aboriginal women and infants to identify those with poorer outcomes so health services and clinicians could target service delivery and improvement interventions at the regional and local levels.	
**Study of out of hospital births:** audit of 32 records of women who birthed locally, detailed field notes, stories collected and unstructured interviews with 7 locally birthing women and 5 of their family.	Influenced system improvement and the establishment of the MGP.	Honours student graduated and now doing a related PhD in remote Aboriginal Australia; 1 publication.
**Parenting study:** Longitudinal interviews and observations with 15 women from each field site from pregnancy until their babies were 12 months. Discussions were held with women and family members and narratives collected.	Repeated requests for conference presentations (including 2 keynotes at national conferences) and staff seminars to access and discuss this work and the implications it has on the delivery of health and human services.	Development of research skills in Aboriginal co-researchers; 1 publication.
**Impact of colonisation of health care in the NT;** An Aboriginal PhD candidate with Aboriginal co-researchers in a candidate led study of the quality and nature of health care with a case study on intergenerational learning about birthing.	Evaluation of cultural security across general health services conducted as an industry sponsored piece of research that fed into policy and services with a case study analysis in train sponsored by the candidate.	PhD in train; 1 publication, one book chapter and one eBook chapter on basis of method used; Industry Case Study Report on Cultural Security.
**Post intervention evaluation:** 66 participants were interviewed, record audit repeated, field notes kept and observations undertaken in remote sites as well as RDH replicating baseline data collection.	Published in a commissioned report for the NT Government with papers nearly completed for publications.	Contributing to PhD student C- well advanced in thesis. Reports (n = 2) to Industry. Publications in preparation.
**Participatory Action Research Study;** Baseline data on problems with transfer of information between the RC and remote clinics led to study between senior manager and 2 researchers on improving the system.	Work between a CI, PhD student and senior manager on starting to improve the system made progress towards improvement, particularly in discharge planning.	Contributed to PhD student C’s doctorate; I publication.
**Costing study:** 315 mothers and singleton infants who were clients of the MGP compared with 408 mothers with singleton pregnancies from the baseline study post MGP intervention. Direct costs from the Department’s perspective from first antenatal visit till 6 weeks post-partum and infant data from birth to 28 days.	Has strengthened the sustainability of the MGP model with a costing paper that demonstrates the new system actually saves money as well as producing better results.	Post-Doctoral researcher trained by the project in health economics led the analysis and publication of this data; 1 publication.
**Benchmarking of neonatal nursery admissions;** Records of all neonates (n:463) born in 2010 and admitted to nursery.	Demonstrated admissions are within national benchmarks for admissions and justified. Study done with paediatrician responsible for nursery care in NT. Confirms baseline and evaluation data.	Paper ready for submission and contributing to PhD candidate D’s thesis.

In brief, we began the health services investigation with a baseline study in the two remote sites and the RC hospital. Data was obtained from 412 mothers and their 413 infants across a trajectory of care from 2004–2006. From this sample a range of sub- studies were conducted (refer to Table 1) that identified patterns of health service utilisation [16,17] as a proxy measure of acceptability, discharge processes [18,19], barriers to care [20], adherence to guidelines on maternity care [20] and infant treatment for anaemia and growth faltering [16,21] as measures of care quality. The women’s experience also informed our analysis of ‘quality’.

An epidemiological investigation of 7,560 mothers with singleton pregnancies from the routinely collected NT perinatal data set for NT births occurring between 2003–5 was conducted. Univariate and multivariate methods examined the impact of Aboriginal status, remoteness and quality of care and age on maternal and infant outcomes for NT births. We also used this epidemiological sub- study to investigate the impact of Aboriginal status on different cohorts. We compared data for 700 Aboriginal mothers aged 13–19 years (teenagers) with 1,721 adult Aboriginal mothers (20–34 years), as well as with 254 non-Aboriginal teenagers. Community records, birth registrations and perinatal counts of births occurring in the two fields sites between 2004 and 2006 were compared to examine the accuracy of birth counts for the field sites.

In order to examine complex health and social phenomena in a group of women and families that influence health service delivery, the ARC funded ethnographic work interviewed participants and accessed a small number of clinical records (See Table 1) [22,23]. One study investigated previous ‘out of hospital’ births [24] (n = 32 records and interviews with 7 women and 5 families) and another, a longitudinal design set in both study sites, evaluated women from pregnancy till their infants were 12 months (n = 12), including their and their family’s experiences of early parenting [15].

## Results

These sub- studies provide an overall picture of the standard of maternity or infant care delivery. They describe women’s experiences with services and cultural practices in relation to parenting, and identify systemic barriers to quality and non-adherence to evidence based guidelines. The studies additionally identified local indicators [25-27] (based on routine data sources and our baseline data) that could more precisely inform the measurement of performance.

### The state of maternity care delivery and maternal health

Baseline data from record audits revealed ANC occurred frequently for Aboriginal women, in excess of recommended guidelines. It began late however, not within the recommendations of ANC in relation to trimesters of pregnancy. Records described poor quality of care and poor adherence to evidence based guidelines in several key areas e.g.: smoking cessation advice, treatment of anaemia, urinary tract infections (UTI) and sexually transmitted infections [16]. These factors are all associated with preterm birth, LBW and high hospitalisation rates of neonates. In our sample, one third of the neonates were admitted to neonatal intensive care, following birth [17].

Inappropriate resourcing of remote health services, poor co-ordination, lack of continuity of midwifery carer, as well as discriminatory attitudes and poor practices of clinicians influenced the delivery of ANC [20]. High levels of complications were common: 23% had antenatal hospital admissions, 50% of women were anaemic (Hb <11.0 g/dl), 45% had abnormal urine test results indicating UTI, 21% had preterm labour resulting in preterm birth, and 22% were recorded to have sexually transmitted infections [20].

Baseline data showed 10% of births occurred in the remote communities despite guidelines recommending all women be transferred for birth. Just over one-third (36%) of these were preterm births [20]. The first small ethnographic study identified women’s decision to birth locally rather then be transferred. This decision was influenced by their previous frightening experiences when giving birth in the RC, their assessment of risk in relation to their own health status and their responsibilities to other children [24].

Following birth, communication and handover of care from clinicians at the regional hospital to the staff of the remote health centres was inadequate. Observations and interviews showed discharge summaries were late or absent and many inaccuracies were identified [19]. Poor documentation, communication and co-ordination between hospital and health centre staff and a lack of clinical governance compromised the standard of care during the discharge and afterwards. After discharge, care was fragmented without qualified child and family health nurses working in a supportive role with parents. A participatory action sub-study led by a doctoral student with health service staff showed some improvements to the discharge process [18] with better coordination of communication.

By six months postpartum, 45% of women had documented postnatal morbidities such as anaemia (20%), UTI (8%) and reproductive tract infections including caesarean section wound infections (8%), and 8% required hospital admission. Most women accessed remote health services at least once, however few had documented postnatal care [16].

In response to these findings (and funding opportunities resulting from the Northern Territory Emergency Intervention) [28] the Midwifery Group Practice (MGP), a new model of care, was introduced.

#### ***The intervention: the midwifery group practice (MGP)***

The MGP was staffed by midwives, Aboriginal Health Workers (AHWs), Aboriginal midwifery students and an Aboriginal ‘senior woman’. This became the main health service-led ‘intervention’ of the study, evaluated and reported elsewhere [29,30]. Designated full- time midwifery positions in each of the communities were also introduced. Attempts were also made to improve infant care during this period, including increasing the staffing of ‘well baby’ services in both communities and introducing a training program that many nurses took ‘on-line’ to improve their knowledge in child health and development.

This intervention provided women from the two remote communities with continuity of care from a primary midwife, assigned at the time the women were in the RC hospital. Designated midwives (with no routine nursing duties) were also introduced in five remote communities, including the two field sites [31].

### How did the MGP impact on maternal and infant health?

Significant improvements were made to health services including statistically significant improvements in maternal record keeping; ANC (fewer women had no ANC and more had >5 visits), antenatal screening tests and smoking cessation advice; a reduction in fetal distress in labour; and, a higher proportion of women received postnatal contraception advice [30].

The experiences of women, midwives and others during the establishment and the first year of the MGP also were reported positively [31]. Furthermore, women’s engagement with the health services through their midwives improved. Midwives in the RC were regularly receiving text messages reporting baby weights many weeks after birth. Quality of care was enhanced and a number of positive clinical outcomes were found. A cost consequence analysis showed cost savings were found as a result of significantly reduced birthing and neonatal nursery costs [29].

#### ***What did not change and why?***

Our evaluation in 2012 [30] showed further improvement in clinical care is still needed. Some adverse health conditions appeared to increase, possibly due to improved documentation. A paper (in preparation) describes in more detail results than have so far been published from the report of the MGP evaluation [30].

Most contextual factors seemed to have been addressed by the introduction of the MGP. Appropriately qualified staff is now providing continuity of care. The reported absence of Aboriginal leadership is being better addressed through improved relationships between midwives and their clients.

### The state of infant care delivery and infant health

By one year of age 59% of infants in baseline studies were admitted to hospital at least once with the rate of hospitalisation per infant year 1.1 (95% CI 0.9-1.2) [17]. At baseline, one-third of infants were admitted to the neonatal nursery at birth, predominantly for prematurity. Infants presented to the remote health centre on average 28 times by age one [17]. Half of these were for respiratory, skin and gastrointestinal symptoms with the remaining presentations for review or routine care. Sixty-eight percent of infants were anaemic before age one and 86% suffered growth faltering [21]. Clinical identification, management and treatment completion was poor for both growth faltering and anaemia in remote health centres [17].

Both health centres had high staff turnover and limited capacity to provide continuity of care between staff and parent [21]. Most staff had no formal qualifications in child and family health resulting in limited knowledge and skills in infant health and development. Furthermore they worked in poorly structured and fragmented services. Hospitalisation of neonates at birth was significantly higher than expected in the baseline data when compared to national figures (e.g. preterm birth <37 weeks; Baseline: 21% (2004–06) [16] vs 13.7% (Australian Aboriginal, 2006) and 5.1% (Australia non-Aboriginal 2006) [32]. Given the rate and acuity of presentations, along with our data on infants that had already been hospitalised as neonates and our epidemiological evidence on birth rates [26], staffing was clearly inadequate and compared poorly with the improved model of maternity care [18,31].

Both communities demonstrated the impact of poor engagement with Aboriginal community leaders, community services, absence of AHWs and of Aboriginal leadership in the way services were designed, delivered or ‘owned’ by their local community. There was insufficient time for health promotion [21]. Qualitative analyses of the 2012 evaluation data showed no improvements in infant health service provision [30], however detailed analysis (unpublished) of quantitative and qualitative outcomes for infants is ongoing.

#### ***What factors might have influenced these findings?***

##### 

**Deficiencies in the quality and availability of data to inform decisions about the provision and delivery of services** Epidemiological analyses of 2003–2005 NT perinatal data quantified inequalities between groups of Aboriginal women and newborns Aboriginal women and infants have worse outcomes if they live remotely, especially if they live in the Top End [6]. This sub-study confirmed existing evidence that the prevention of smoking during pregnancy and the delivery of high-quality ANC are needed to improve Aboriginal MIH outcomes. When different Aboriginal age cohorts were analysed, Aboriginal mothers < 20 years were more likely to have a normal birth than Aboriginal mothers aged 20–34 years.

We compared the numbers of births for our sites using community birth records, birth registrations and the midwives’ data collection [26]. Birth registration and perinatal data sets appear to underestimate birth counts when verified locally with community birth records. The range is notable at the local level (ten infants or more identified in both settings) and can lead to an underestimation of workload, resources needed and intensify already existing staffing pressures. Mobility of Aboriginal women (an integral part of Aboriginal life) plays an important part in the changes in population numbers and should be catered for in routinely collected NT data [26].

##### 

**Discrimination and inadequate understanding about Aboriginal culture** We explored the experiences and beliefs of families, in an ARC funded sub- study, as they cared for their infants between birth and their first birthday. Stories were collected about child rearing, development, health and well-being. This research showed major discordance between Western and Aboriginal views about children's agency and role within the family [15].

Unintentional discrimination and racism were also evident in our observations and interviews across the program of work in remote and regional centre fieldwork [23]. The invisibility of Aboriginal leadership seems to contribute to this [17]. The new MGP model appears to have reduced this to some degree with data suggesting a change in culture in the RC maternity wards; possibly due to the relationship-based advocacy role that was played by the midwives and Aboriginal workers in the new model [30].

##### 

**Development of training opportunities and research capacity** An additional goal of our work was to engage Aboriginal health workers and communities in research with a view to improving Aboriginal care delivery and health outcomes [13,33]. Our program of work made an important contribution to the MIH workforce and development of research skills (See Table 1). Researchers undertaking doctoral or other research degrees conducted many of the sub-studies as they simultaneously developed research skills desirable for professionals living and or working with remote and Aboriginal populations.

## Discussion

This paper describes MIH care at two different points in time and reports the results of a collaborative health service redesign and a program of research aimed at health system reform. Researchers have worked alongside the NT Department of Health as they have developed policies and led reforms. The partnership between the research team and the Department of Health enabled evidence-informed improvements to health service delivery to be made. Our response to industry partners is exemplified by the latest sub-study conducted at the request of a leading NT clinician into the reasons for neonatal admissions to the nursery to benchmark and compare these nationally and clarify that these high rates of admission were all necessary (paper under review). Further publication and analysis of services conducted in the final year, with a focus on evaluation of improvements, is in train. Some reform has been successful, such as changes to models of maternity care showing positive outcomes [30], however improvements to infant care are urgently required.

Our program of work has identified continuing problems with infant service delivery that should be addressed at the institutional level. Practices such as adhering to evidence-based guidelines, attaining accurate enumeration of births for remote areas and organising staffing and skills around known workload and morbidity levels need to be implemented. The sub-study of service utilisation observed that one-third of neonates from the communities were admitted to the special care nursery [17] at rate more than twice the national average [3]. Aboriginal infants are already compromised at birth with a birth weight nearly 186 g less than other Australian babies [3], more so if the mother smokes (nearly 250 g lighter) [34]. The qualifications required of staff working in this area must be addressed institutionally as almost all nurses working with infants and children were insufficiently prepared to work in this specialised area [21]. Improvement is also needed to the organisation and delivery of infant and child health care with a more supportive and continuous relationship with parents a priority.

It seems that teenage pregnancy is common in the NT, however problems usually associated with Aboriginal teenage births (such as LBW) are not due to maternal age, but rather related to the underlying poor health, socioeconomic disadvantage and a system that is challenged to support these young women, both culturally and medically. There is a need to use specific indicators, drawn from routinely collected data and our research, to inform and more accurately reflect the performance of health services and Aboriginal health status in this setting [25-27].

Our remote fieldwork identified a service that is dominated by acute presentations, rather than a community-engaged primary health system that promotes community, family health and well-being. Health system deficiencies in knowledge, recognition and support of local culture and child-raising need remedying to allow staff to be effective in promoting health and building resilience with parents of vulnerable infants [15]. Reforms to service design such as extending the continuity of care model now employed by designated midwives and the MGP into child health would enable staff to work alongside women and families. Similarly, respectful informed relationships with community leaders, focused on parenting and working together, could address the seriously inequitable outcomes of Aboriginal infants.

Coordination of care between remote and regional services, particularly information sharing and communication, remained poor but is improving with the use of electronic information systems relatively recently introduced. Further improvement could occur by making MGP midwives responsible for discharge summaries with phone handovers (prior to discharge) to the care provider in the community. Computer based information systems for MIH could monitor adherence to clinical guidelines centrally, highlight abnormal health findings and encourage appropriate responses from remotely located care providers. This is particularly important in infant care and child health where staff lack formal knowledge and skills considered essential for employment in other areas of Australia.

Funding of current services is an additional challenge. There are very few universal health care payments or Medicare reimbursement items that the NT government can claim against to gain refunds from the federal government for providing these services. In addition there are very few private practitioners working in remote Australia who can generate additional Medicare revenue [35]. Eligible midwives, who can claim against the national insurance system, are also currently underutilised in remote areas and, as with general practice, the relative numbers of uninsured and poor clients make a private practice model problematic except in very large remote communities.

### Limitations

Broadly based programs of work that aim to measure and describe the complex ‘real world’ of health services behavior and system improvement pose significant challenges in terms of designing, synthesising and reporting the results. A mixed-methods approach has enabled us to grapple with this complexity. Scrutiny of design occurred through highly regarded peer-reviewed funding mechanisms. The methodological rigour of our designs, and therefore the validity of the results of this synthesis are further demonstrated by the peer-reviewed publications from each sub- study.

## Conclusion

This program of health services-oriented research identified serious deficiencies in the quality of MIH services for Aboriginal families living in remote communities in the Top End of Australia. Significant improvements were achieved. This has occurred through the engagement and support of Aboriginal women, policy makers, clinicians and managers who participated throughout the program of work and who are still working with our findings. Translation is still occurring.

The introduction of designated midwives and implementation of the MGP has resulted in improved clinical effectiveness and quality of care. This appears to be a sustainable, cost-effective model that should be rolled out to all women relocating for birth [29]. With one-third of Aboriginal and Torres Strait Islander birthing women living in remote Australia this strategy could be a significant contribution to ‘closing the gap’. In order to address the ongoing pattern of substandard care for infants and rates of preventable adverse infant health outcomes, more work is needed by health services, particularly in terms of targeting system redesign and the existing level of skills. This requires a commitment to address these issues at the institutional and service delivery levels by staffing according to volume of need, and providing staff with the skills necessary to conduct the work. Importantly, these reforms should be conducted in a system designed to engage Aboriginal leaders in a primary health and ‘well-being’ oriented model of care.

## Competing interests

The authors declare that they have no competing interests.

## Authors’ contributions

LB conceived the synthesis of the 1 + 1 Program of research and drafted the manuscript. MS, SBZ, SKr, SKi, CJ, SB, TD and YG contributed to subsequent drafts of the manuscripts. LB, MS, SBZ, SKr, SKi developed the summary table of research. CWN and MW provided Aboriginal guidance and scrutiny of methods, in addition to cultural and local advice for researchers. Each author was involved in at least one of the sub-studies of the 1 + 1 Program of research. All authors, apart from MW (deceased) read and approved the final manuscript.

## Pre-publication history

The pre-publication history for this paper can be accessed here:

http://www.biomedcentral.com/1472-6963/14/241/prepub
